# Leukemia Mediated Endothelial Cell Activation Modulates Leukemia Cell Susceptibility to Chemotherapy through a Positive Feedback Loop Mechanism

**DOI:** 10.1371/journal.pone.0060823

**Published:** 2013-04-01

**Authors:** Bahareh Pezeshkian, Christopher Donnelly, Kelley Tamburo, Timothy Geddes, Gerard J. Madlambayan

**Affiliations:** 1 Department of Biological Sciences, Oakland University, Rochester, Michigan, United States of America; 2 Radiation Oncology, William Beaumont Health System, Royal Oak, Michigan, United States of America; INRS, Canada

## Abstract

In acute myeloid leukemia (AML), the chances of achieving disease-free survival are low. Studies have demonstrated a supportive role of endothelial cells (ECs) in normal hematopoiesis. Here we show that similar intercellular relationships exist in leukemia. We demonstrate that leukemia cells themselves initiate these interactions by directly modulating the behavior of resting ECs through the induction of EC activation. In this inflammatory state, activated ECs induce the adhesion of a sub-set of leukemia cells through the cell adhesion molecule E-selectin. These adherent leukemia cells are sequestered in a quiescent state and are unaffected by chemotherapy. The ability of adherent cells to later detach and again become proliferative following exposure to chemotherapy suggests a role of this process in relapse. Interestingly, differing leukemia subtypes modulate this process to varying degrees, which may explain the varied response of AML patients to chemotherapy and relapse rates. Finally, because leukemia cells themselves induce EC activation, we postulate a positive-feedback loop in leukemia that exists to support the growth and relapse of the disease. Together, the data defines a new mechanism describing how ECs and leukemia cells interact during leukemogenesis, which could be used to develop novel treatments for those with AML.

## Introduction

Annually, greater than 12,000 new cases of acute myeloid leukemia (AML) are reported with <10% of these achieving disease-free survival and the majority of patients (∼80%) relapsing despite initial remission [1]. To overcome these bleak outcomes, a better understanding of how leukemia cells survive therapy must be developed. Normal blood formation involves carefully orchestrated interactions between hematopoietic stem cells (HSCs) and extrinsic signals mediated via 'niches' located in the endosteal and vascular regions of the bone marrow [2], [3], [4]. The mechanisms through which the endosteal niche affects leukemia progression are now being defined [5], [6], however; the effects of the vascular niche remain obscure and will require much investigation in the coming years [7], [8].

Studies have demonstrated a supportive role of endothelial cells (ECs) in normal hematopoiesis both *in vitro* and *in vivo*. During *in vitro* culture, ECs maintain the repopulating potential of HSCs and protect bone marrow (BM)-derived CD34^+^ cells from ionizing radiation [9], [10], [11], [12]. *In vivo*, it was reported that EC infusion following total body irradiation conferred accelerated recovery of BM sinusoidal vessels, BM cellularity, and peripheral blood neutrophils and platelets as well as radioprotection of stem and progenitor cells concomitant with a 4.4-fold increase in BM HSCs following irradiation [13], [14]. Without ECs, irradiation caused a disruption of the BM vasculature and cellularity, ablation of HSCs, and pancytopenia in control mice. This data demonstrated that ECs create BM microenvironments that augment hematopoiesis and suggest a relationship between ECs and normal hematopoietic reconstitution *in vivo*. The fact that phenotypically defined HSCs are readily found near sites of sinusoidal endothelium in the spleen and BM provide further support for the intercellular modulating effects between these cell types and, in fact, the synergistic role of the endothelial/vascular niche in hematopoiesis is now well documented [2], [4].

A similar intercellular relationship may exist in leukemia. Clinically, increased levels of angiogenesis have been directly observed in patients with AML providing strong indications for a role of ECs in the propagation of leukemia [15]. In these studies, BM samples stained for EC markers showed that blood vessel density was significantly higher in AML versus normal BM samples. The findings that there are an increased number of circulating ECs in the peripheral blood of AML patients are further suggestive of increased EC activity [16], [17]. Interestingly, it was reported that patients achieving complete remission following induction treatment showed a significant reduction in the numbers of circulating ECs in comparison to pre-treatment values [18]. Together, this clinical data suggest that ECs are an active component of leukemia.

Similarly, *in vitro* experiments have demonstrated the ability of ECs to enhance the proliferation of AML blast and progenitor cells [19]. To show that ECs support leukemia *in vivo*, we previously performed studies in which immunocompromised mice engrafted with human AML of varying subtypes were treated with the vascular/endothelial disrupting agent OXi4503 [20]. This EC targeting strategy resulted in phenotypic and molecular elimination of human leukemia in mice including those with activating mutations in FLT3 ITD. Similar results have been observed using combretastatin-A4-phosphate [21]. The fact that anti-vascular agents were able to diminish leukemia in these systems provided, for the first time, direct evidence of a supportive endothelial contribution to leukemia *in vivo*, which could be circumvented through a concerted EC targeting therapy.

While the role of ECs in leukemia seems likely, what is lacking is the mechanistic understanding of the types of interactions that occur and, just as important, the factors leading to the development of these interactions. Interestingly, leukemia cells possess the ability to alter BM microenvironments as well as EC behavior. For example, *in vitro* co-culture of ECs with human AML altered EC behavior in a non-cell-autonomous manner resulting in increased EC proliferation [22], [23], [24]. Others have shown that transplanted leukemia cells can disrupt BM niche activity *in vivo* resulting in abnormal microenvironments [25]. Upon engraftment in these regions, normal CD34^+^ hematopoietic progenitors exhibited lower proliferation and the inability to mobilize into circulation. This same group used *in vivo* confocal imaging to demonstrate that leukemia cells preferentially home to unique E-selectin expressing ECs [26]. Given the close association of these cells *in vivo*, this data collectively suggests that ECs and leukemia cells can dramatically impact one another.

These findings indicate an intimate relationship between ECs and leukemia, wherein leukemia cells are able to significantly alter EC activity. Given this response, we hypothesized that leukemia cells alter EC activity, and through this altered activity, ECs produce microenvironments responsible for leukemia growth, survival and, ultimately, relapse. In this paper, we describe the ability of leukemia cells to change the behavior of resting ECs by inducing the biological process of EC activation. EC activation occurs in response to a wide range of inflammatory factors leading to altered cell morphology as well as increased expression of cell adhesion molecules, such as E-selectin, and production of various cytokines [27]. We demonstrate that in this activated state; ECs participate in leukemogenesis by orchestrating a series of steps that affect the progression and relapse of the disease. Overall, this data implicates EC activation as a new mechanism in leukemia, demonstrates that processes involved in the inflammatory response are active in leukemia and highlights the potential of using anti-inflammatory drugs during standard treatment of the disease.

## Materials and Methods

### Cell Culture

#### Cell Lines

For these studies, KG-1 (M0) and HL-60 (M3) human leukemia cells lines (ATCC, Manassas, VA, USA) and human umbilical vein endothelial cells (HUVECs) were used (Lonza, Walkersville, MD, USA). Cells were cultured using the EGM-2 MV Bullet kit (Lonza). For all experiments, HUVECs (50,000-100,000 cells/well) were cultured in 12 well collagen-coated plates and incubated in 5% CO_2_ at 37°C for 2–3 days to reach desired confluence. KG-1 and HL-60 human leukemia cells lines were chosen as they represent different AML subtypes (KG-1/early myeloblasts/M0 and HL-60/promyelocytic/M3) and AMLs with differing treatment strategies (KG-1/induction and consolidation chemotherapy and HL-60/ATRA therapy). These cells were maintained in Iscove’s Modified Dulbecco Medium (IMDM; Hyclone, Fisher Scientific, Hanover Park, IL, USA) plus 20% FBS (Hyclone, Fisher Scientific). KG-1 and HL-60 cell lines were maintained and passaged following ATCC guidelines.

#### Primary Cells

These studies also tested the effects of EC activation in leukemia using primary ECs derived from human bone marrow (Lonza). Primary ECs were grown as previously described [28] with only slight modifications. Briefly, bone marrow cells were suspended in EGM-2 medium and plated in culture dishes coated with type 1 rat-tail collagen (BD Biosciences, Bedford, MA, USA) at a density of 5×10^6^ to 1×10^7^ cells per well. After 24-hours, non-adherent cells were removed and the remaining adherent cells fed with fresh EGM-2 medium. Medium was changed every day for the first 7-days and then every other day until a confluent layer of ECs was generated. Primary human bone marrow mononuclear cells (5×10^6^ per co-culture) were used as controls to show that normal blood cells do not induce EC activation and that this process was specific to leukemia cells. The mononuclear cell fraction was collected with Ficoll Paque (Amersham Biosciences, Piscataway, NJ, USA) centrifugation purification.

#### Co-Cultures

Co-cultures were established to examine intercellular interactions between ECs and leukemia cells. Here, primary ECs or HUVECs were first grown then 1×10^6^ - 5×10^6^ KG-1 or HL-60 cells were added either in direct contact or separated by a 0.4μm transwell insert (non-contact cultures). Co-cultures were maintained in EGM-2 media with 10% FBS unless otherwise described.

### Endothelial Cell Activation

To induce EC activation, primary ECs or HUVECs were co-cultured with 1×10^6^ - 5×10^6^ KG-1 or HL-60 cells in direct contact or separated by a transwell. Cells were then trypsinized and stained for E-selectin (clone 68-5H11, APC), CD105 (clone 266, PE) and CD45 (clone 2D1, APC-Cy7 and clone HI30, APC; all antibodies from BD Pharmingen, San Jose, CA, USA) then analyzed by flow cytometry using a FACSCanto II (BD Biosciences) to confirm EC activation. Secondary activation of resting ECs was achieved using a similar method. As positive controls, primary ECs or HUVECs were activated with 10-20ng/mL TNF-α (Humanzyme, Chicago, IL, USA). The expression of the E-selectin binding ligand, sialyl Lewis^X^ (CD15; Clone HI98, FITC; BD Pharmingen), on the surface of cells was also analyzed by flow cytometry.

### Cell Adhesion Study

Previously activated (with TNF-α) or non-activated ECs were washed twice with PBS and 1×10^6^–5×10^6^ KG-1 or HL-60 cells were added in fresh growth media. After 30 minutes at 37°C, non-adhered cells were removed with PBS, collected and enumerated. Adherent cells were trypsinized and re-suspended in PBS with 2% FBS. These cells were subsequently stained for CD45, CD105 and E-selectin and analyzed by flow cytometry for further leukemia cell enumeration as well as to characterize and confirm EC activation.

### Analysis of Proliferative Status

Analysis of proliferative status was through BrdU uptake following the manufacturers protocol (BD Biosciences). Briefly, 1×10^6^–5×10^6^ cells were incubated in 10 µM final concentration BrdU in growth media for 35 minutes. Leukemia cells were then stained with anti-CD45 and anti-BrdU (clone 3D4, FITC) antibodies for 15-20 minutes and analyzed by flow cytometry.

### Chemotherapy Treatment and E-selectin Blocking Studies

Contact co-cultures with KG-1 and primary ECs or HUVECs were prepared to activate and induce cell adhesion. Cultures were then treated with 200 µM cytosine ß-D-arabinofuranoside (Ara-C, Sigma-Aldrich, Saint Louis, MO, USA) or 0.1 µM idarubicin (IDA, Sigma-Aldrich) for 24 hours. Non-adherent and adherent cell populations were collected separately and stained with combinations of CD45, CD105 and Annexin V (FITC, BD Pharmingen) to assess the effects of chemotherapy by flow cytometry.

The effects of chemotherapy treatment on leukemia cells that were prevented from adhering to activated ECs were assessed using an E-selectin blocking strategy. Here, HUVECs were activated with KG-1 co-culture or with 10-20ng/mL TNF-α in the presence of 50 µg of anti-E-selectin antibody (clone BBIG-E4 and clone BBA26, R&D Systems, Minneapolis, MN, USA). Following TNF-α activation only, ECs were washed with PBS and 1×10^6^ KG-1 cells were added in growth media supplemented with an additional 50 µg of anti-E-selectin antibody for 30 minutes. The levels of leukemia cell adhesion were then assessed. Next, 200 µM Ara-C was added and the effects of the blocking strategy on apoptosis measured by staining both non-adherent and adherent cells with CD45, CD105 and Annexin V followed by flow analysis.

To determine if inducing leukemia cell release from activated ECs would increase overall cell killing following chemotherapy, contact co-cultures with KG-1 cells were established. After vigorously removing non-adherent cells, 75 µg of E-selectin blocking antibody was added to each well and incubated for 24 hours to induce cell release as previously described [29]. Next, 200 µM Ara-C was added to each well and incubated for another 24 hours. Adherent and non-adherent cells were tested for apoptotic response as described.

### Statistics

Statistical differences were calculated using Student *t* test. The reported values represent the mean±SEM. A *p* value ≤ 0.05 was considered to be significant.

## Results

### Leukemia cells are capable of activating resting endothelial cells

**Figure 1:**
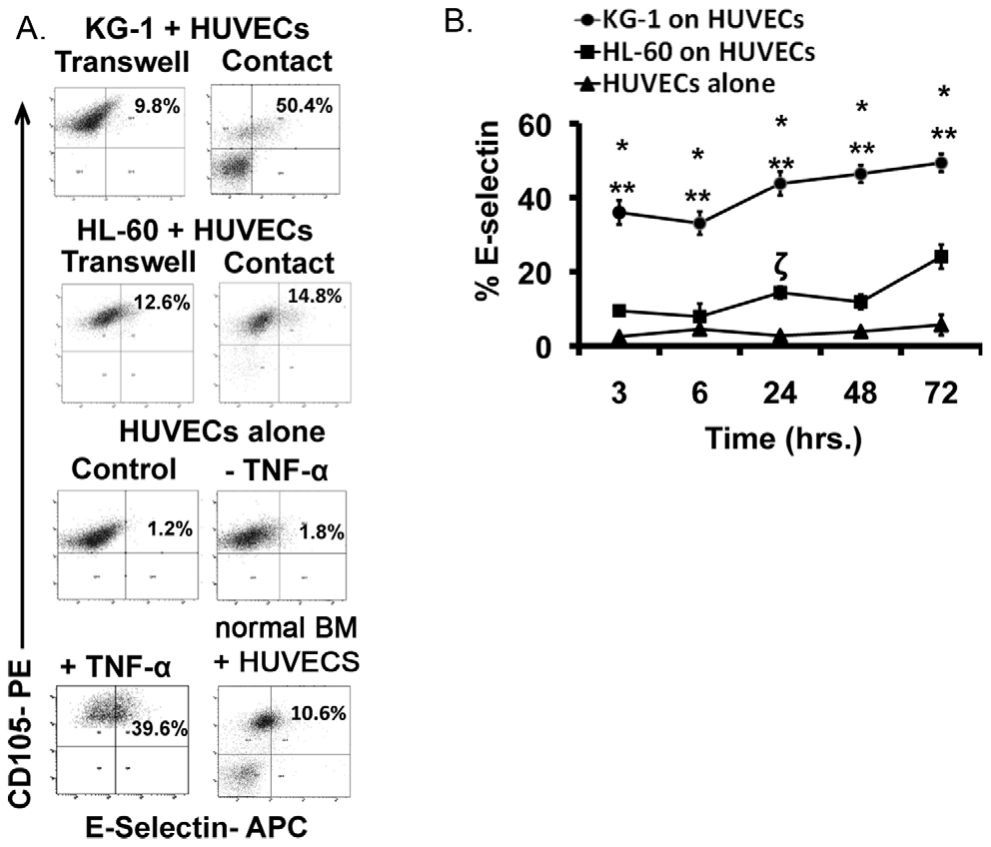
Leukemia cells activate ECs. (**A**) Representative flow plots show levels of EC activation based on percent E-selectin expression specifically on CD105^+^ ECs. Contact and non-contact (transwell) co-cultures of KG-1 and HL-60 on ECs were tested. Representative flow plot of ECs treated with 10ng/mL TNF-α as positive control is also shown as well as plots of untreated ECs and ECs cultured with normal BM. (**B**) The levels of E-selectin expression on ECs activated with KG-1 and HL-60 cells in contact co-culture were determined over a 72-hour time period. * p<0.05 compared to HL-60 on ECs; ** and ζ p<0.05 compared to ECs alone. Gates were established using CD105 stained resting ECs and isotype controls.

To study the ability of leukemia cells to activate resting ECs, co-cultures of HUVECs with KG-1 and HL-60 leukemia cell lines were established. These cells represent distinct AML subtypes with varying modes of treatment (see Materials and Methods). Given the heterogeneity of AML, initial studies were conducted to determine if these different AMLs would induce differing levels of activation. Direct contact and non-contact (separated by a 0.4μm transwell) cultures were tested. As positive controls, HUVECs were treated with 10ng/mL of TNF-α, a cytokine known to induce EC activation [30], [31]. E-selectin (CD62E) is a known biomarker of EC activation [27]; therefore, this cell surface marker was used to quantify levels of EC activation using flow cytometry. E-selectin levels were found to remain constant at 2.5±0.7% for ECs that were cultured alone (Figure 1A). However, 3-hour co-culture resulted in increased E-selectin levels with both KG-1 and HL-60 cell lines (Figure 1A). Notably, the levels of EC activation were higher when cells were grown in contact co-culture suggesting that direct contact exacerbates the activation response. Furthermore, KG-1 cells showed a significantly higher propensity to activate ECs in comparison to HL-60 cells (Figure 1B). Control cultures wherein ECs were exposed to TNF-α demonstrated increased E-selectin expression as expected (Figure 1A). We also observed that normal BM cells induced a slight increase in E-selectin levels to 10.4±1.5% (Figure 1A). This data demonstrates the ability of leukemia cells to activate resting ECs to varying degrees through direct intercellular interactions.

### Endothelial cell activation results in enhanced leukemia cell adhesion

**Figure 2:**
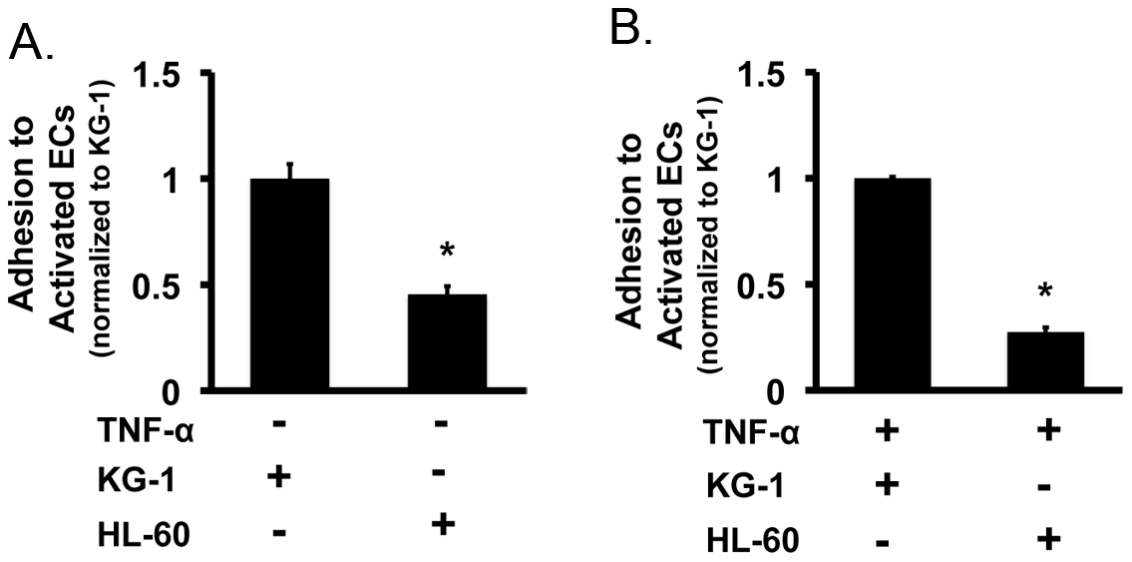
Activation results in the adhesion of leukemia cells to EC layer. (**A**) The levels of KG-1 and HL-60 leukemia cell adhesion to activated ECs were determined following a 3-hour incubation. Values were normalized to KG-1 adhesion levels. * p<0.05 (**B**) Adhesion of KG-1 and HL-60 leukemia cells to ECs activated by 10ng/mL TNF-α . Values were normalized to KG-1 adhesion levels. * p<0.05

Direct contact between leukemia cells resulted in the highest levels of EC activation. Interestingly, further qualitative analysis of these cultures demonstrated that a proportion of the leukemia cells strongly adhered to the activated EC layer similar to what occurs during leukostasis [30]. We hypothesized that enhanced cell adhesion may be a consequence of the activation process. To test this theory, we established co-cultures wherein KG-1 and HL-60 cells were grown in direct contact with ECs for 3-hours and levels of cell adhesion were quantified. Our results demonstrated that a sub-set of both KG-1 and HL-60 cells adhere to EC layer; however, KG-1 cells displayed significantly higher adhesion in comparison to their counterparts (Figure 2A). This finding directly correlated to the relative levels of activation induced by each cell line at 3-hours as HL-60 cells showed a decreased ability to activate ECs in comparison to KG-1 cells (Figure 1B). To further demonstrate that adhesion occurred as a direct consequence of EC activation, we also established co-cultures wherein KG-1 and HL-60 cells were cultured in contact with ECs that were previously activated with 10ng/mL TNF-α for 3-hours. We found that TNF-α activated ECs indeed facilitated adhesion of both KG-1 and HL-60 leukemia cells (Figure 2B). Interestingly, HL-60 cells displayed lower adhesion on EC layer. This suggests that HL-60 cells have an overall lower propensity to adhere to activated ECs compared to KG-1 cells. Based on the results, we concluded that EC activation induces cell adhesion and that the level of activation affects the level of adhesion to ECs.

### Adhesion to activated ECs affects the proliferative status of leukemia cells

**Figure 3:**
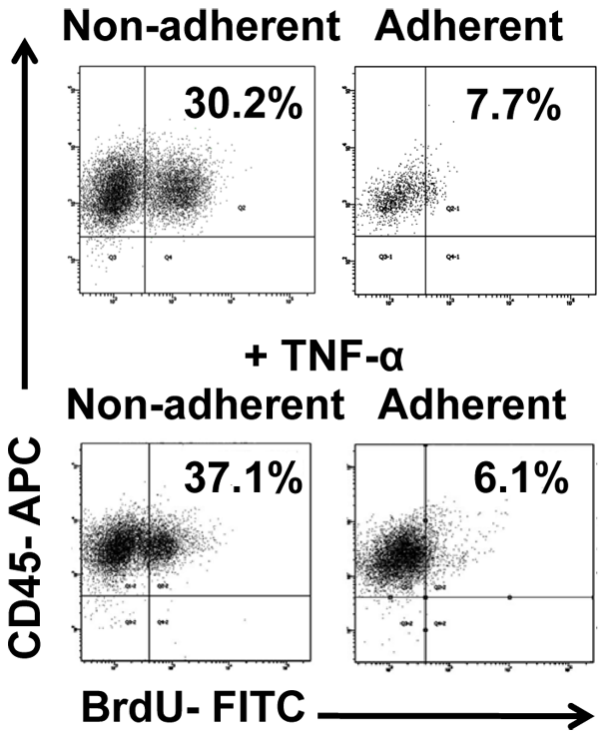
EC activation affects the proliferative status of leukemia cells. Following establishment of co-cultures, representative flow plots show BrdU uptake for non-adherent KG-1. Adherent KG-1 cells are quiescent determined by lack of BrdU uptake (n = 7). Similar analysis was done on leukemia cells co-cultured with TNF-α activated ECs as positive controls. Gates were established using isotype controls.

To begin to elucidate the effects of EC activation, we first assessed how activation affected the proliferative status of leukemia cells. Studies focused on KG-1 cells as these produced the highest levels of activation. Contact co-cultures with KG-1 cells and ECs were prepared and after 24 hours, adherent and non-adherent cells were collected separately. To determine the proliferative status of these cells, each was assessed for their ability to uptake BrdU. In all instances, BrdU uptake was measured in conjunction with CD45 and CD105 surface expression to isolate uptake in leukemia cells and exclude potentially contaminating ECs. Our data convincingly showed that adherent leukemia cells displayed significantly lower levels of proliferation in comparison to non-adherent cells with BrdU levels of 11.7±1.6% and 34.5±1.5%, respectively (Figure 3). Control KG-1 cells cultured in full growth media showed BrdU uptake levels similar to those of non-adherent cells (data not shown). Additional experiments were performed with TNF-α activated ECs with similar results (Figure 3). This data suggests that EC activation-based adherence induced a more quiescent phenotype in comparison to non-adherent cells. This finding showed that EC activation can have a profound effect on the growth of leukemia.

### Leukemia cell adhesion to activated ECs significantly affects their susceptibility to chemotherapy

**Figure 4:**
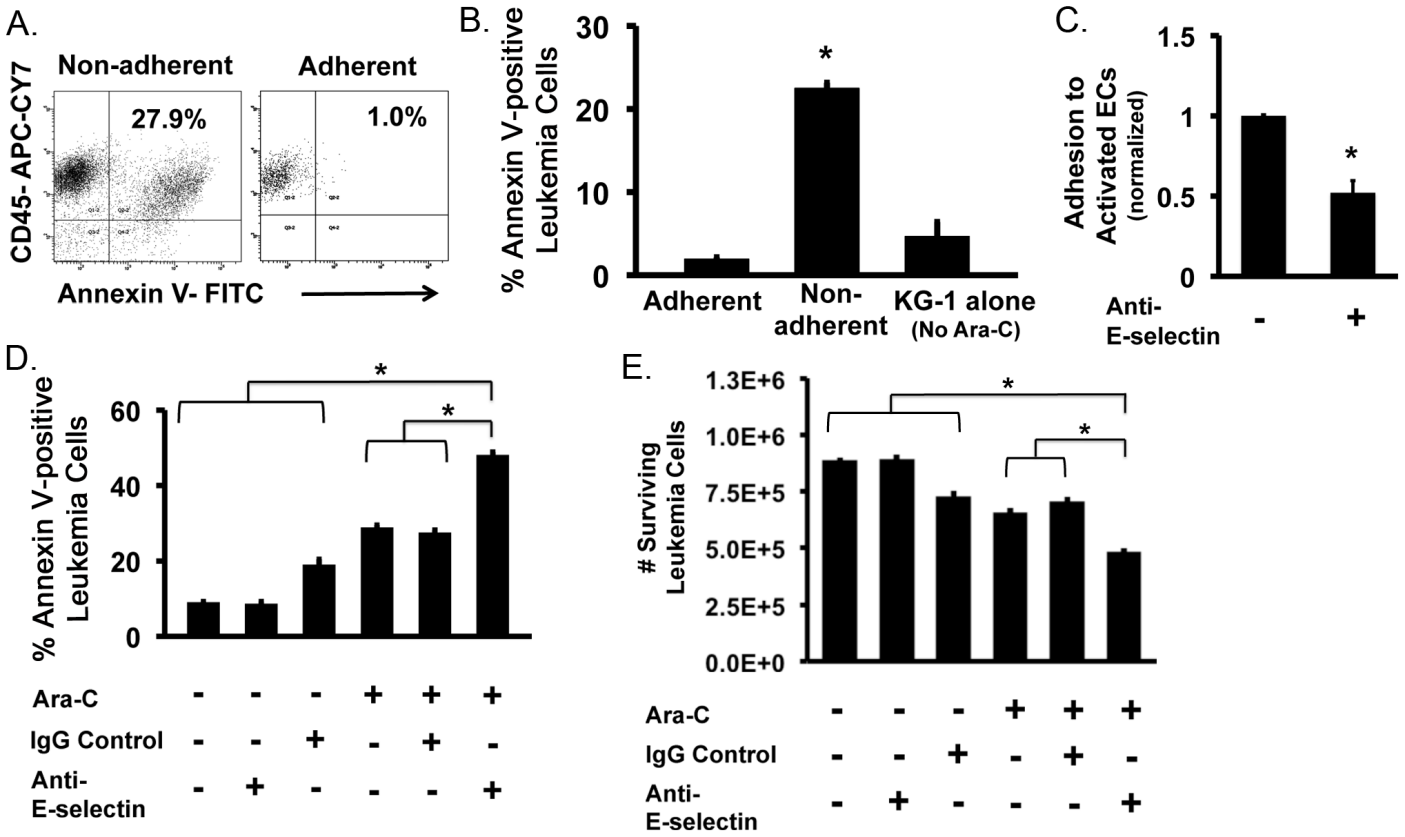
Adhesion to activated ECs affects leukemia cell susceptibility to chemotherapy. (**A**) Representative flow plots of Annexin V staining for adherent and non-adherent populations following treatment with 200 µM Ara-C for 24 hours. (**B**) Percent Annexin V expression on adherent and non-adherent KG-1 cells following incubation with 200 µM Ara-C for 24 hours. The percent Annexin V expression of KG-1 cells without Ara-C was analyzed as a control. * p<0.05. (**C**) Addition of E-selectin blocking antibody to activated EC prior to addition of KG-1 cells significantly (* p<0.05) decreased adhesion. Values were normalized to no anti-E-selectin controls. (**D**) The percent Annexin V staining of KG-1 cells co-cultured on activated ECs with or without Ara-C and/or E-selectin blocking antibody (or IgG control). Incubation with E-selectin blocking antibody enhanced Ara-C mediated apoptosis. Co-cultures treated with irrelevant IgG only were used as controls. * p<0.05. (**E**) Quantitative calculation of the number of surviving (non-apoptotic) leukemia cells remaining with or without Ara-C and/or E-selectin blocking antibody (or IgG control). Anti-E-selectin blocking antibody increased the numbers of cells affected by Ara-C. * p<0.05.

Given the finding that adherent and non-adherent leukemia cells have differing proliferative status, we next looked at the effects of chemotherapy on these populations. Here, we induced adhesion of KG-1 cells on activated ECs via contact co-culture then added 200 µM of Ara-C, a common chemotherapeutic drug used in the treatment of AML [32], and incubated for 24-hours. Adherent and non-adherent cells were separated and stained for CD45, CD105 and Annexin V to isolate the effects of Ara-C on leukemia cells. Our data indicates that adherent leukemia cells are unaffected by Ara-C as most of the cells remained Annexin V negative indicating that adhesion protected these cells from chemotherapy (Figure 4A). Annexin V levels were similar to KG-1 cells not exposed to Ara-C (Figure 4B). In contrast, non-adherent cells showed a marked increase in Annexin V staining demonstrating Ara-C induced apoptosis (Figure 4A and 4B). These findings suggest that adhesion of leukemia cells to activated ECs is a mechanism that serves to protect cells from chemotherapy. In this manner, this protective mechanism may facilitate residual disease that can later serve as cellular modulators of relapsed leukemia.

### Preventing leukemia cell adhesion to activated endothelial cells increases their susceptibility to chemotherapy

E-selectin is known to be up-regulated on activated ECs and serves as a potent biomarker of activation [27]. It has also been shown that leukemia cells can specifically adhere to E-selectin^+^ ECs *in vivo* following transplantation [26]. Therefore, we tested the effects of preventing cell adhesion, using an E-selectin blocking strategy, on the susceptibility of leukemia cells to Ara-C treatment. To robustly test this effect, anti-E-selectin blocking antibody was added during the establishment of activating co-cultures. The addition of E-selectin antibody resulted in significantly fewer leukemia cells adhering to activated ECs (Figure 4C). Subsequent treatment of these same cultures with Ara-C demonstrated that a higher percentage of the total number of leukemia cells were Annexin V positive (Figure 4D). In comparison, cultures treated with only Ara-C or Ara-C plus IgG controls showed lower Annexin V levels suggesting that the blocking antibody treatment augmented the effects of Ara-C. Treatment with blocking antibody alone or irrelevant IgG did not affect cellular apoptosis levels. This evidence suggests that preventing leukemia cell adhesion decreases the protective effects of activated ECs thus increasing total leukemia cell sensitivity to chemotherapy.

To further quantify this effect, calculations were made to determine the numbers of viable (Annexin V negative) leukemia cells remaining following blocking antibody treatment. This analysis demonstrated that prevention of adherence to activated ECs significantly decreased the numbers of viable leukemia cells remaining following Ara-C treatment in comparison to cultures without anti-E-selectin treatment (Figure 4E). Significant decreases were also observed in comparison to controls with no Ara-C treatment. Thus, preventing leukemia adherence to activated ECs represents one mechanism of decreasing overall leukemia burden.

### Adherent leukemia cells that release from activated ECs re-enter cell cycle and are susceptible to chemotherapy

**Figure 5:**
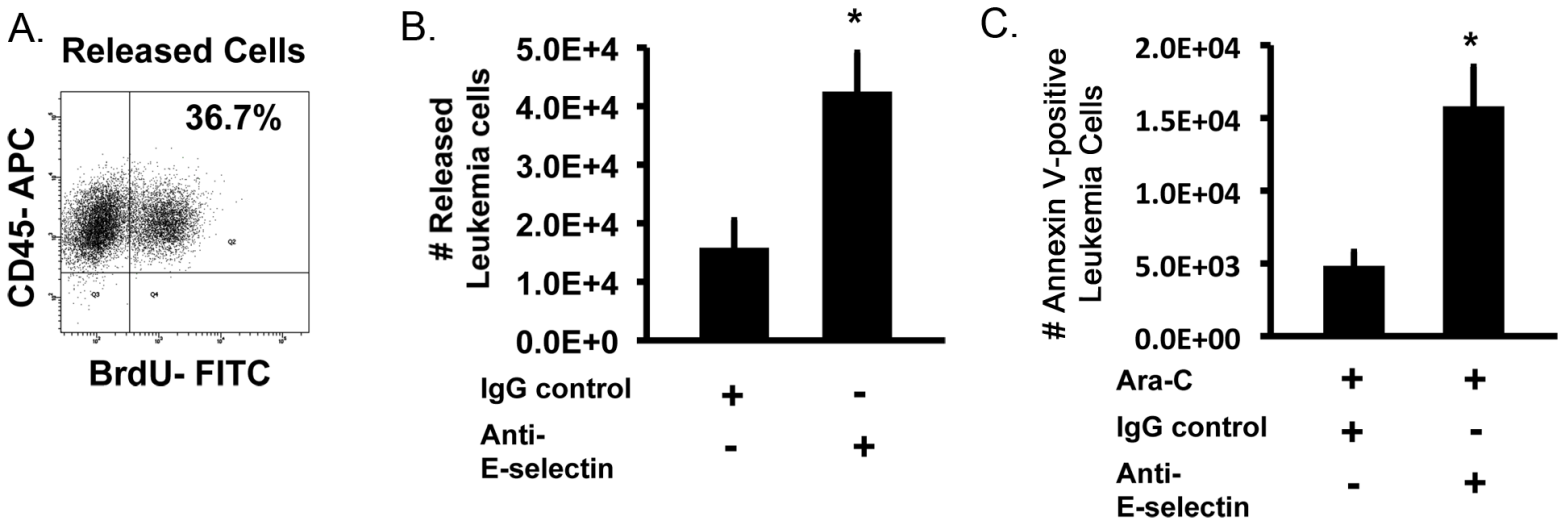
Releasing adherent leukemia cells enhances cell killing by chemotherapy. (**A**) Representative flow plots showing that adherent leukemia cells that survived Ara-C treatment re-enter cell cycle upon release from activated ECs. Cell cycle status was determined based on BrdU uptake. (**B**) Addition of anti-E-selectin antibody to adherent KG-1 cells significantly increased the number of released cells compared to IgG treated controls. (**C**) Adherent KG-1 cells were treated with E-selectin blocking antibody or an irrelevant IgG control and exposed to 200 µM Ara-C. The total number of Annexin V positive released leukemia cells is increased in the presence of anti-E-selectin antibody following treatment. * p<0.05.

We next analyzed the fate of adherent cells in our co-culture systems. This is an important consideration given the fact that leukemia cells are known to regularly attach to ECs following transplantation. By following the fate of adherent cells, we discovered that, following Ara-C treatment and removal of remaining non-adherent cells, some adherent leukemia cells eventually released themselves from activated ECs. Importantly, these newly released leukemia cells were not only viable but also re-entered cell cycle with 23.6±0.7% of cells showing BrdU uptake (Figure 5A). This finding closely mimics what may be observed during leukemia relapse following chemotherapy.

With the finding that adherent cells eventually release and re-enter cell cycle, we next determined if enhancing the release of leukemia cells from activated ECs would also affect overall leukemia cell killing by chemotherapy. We initially tested the ability of our anti-E-selectin antibody to induce this response. Incubation with anti-E-selectin antibody significantly increased the number of leukemia cells released from activated ECs in comparison to IgG alone treated controls (Figure 5B). This result was similar to those observed when anti-E-selectin was used to prevent adhesion in our co-culture systems (Figure 4). Next, we determined if subsequent treatment of these newly released cells with Ara-C would induce apoptosis. Quantitative analysis of the numbers of Annexin V positive leukemia cells demonstrated that anti-E-selectin treatment not only enhanced leukemia cell release but also significantly increased the numbers of apoptotic cells (Figure 5C). Interestingly, the data also showed that 37.2±2.1% of the released cells were Annexin V positive which coincided with the levels of BrdU uptake by these same cells (Figure 5A). Overall, our data indicates that quiescent adherent leukemia cells can be released from activated ECs and become proliferative (i.e. mimicking relapse) making them susceptible to subsequent chemotherapy killing.

### Evidence of a positive feedback loop in leukemia

**Figure 6:**
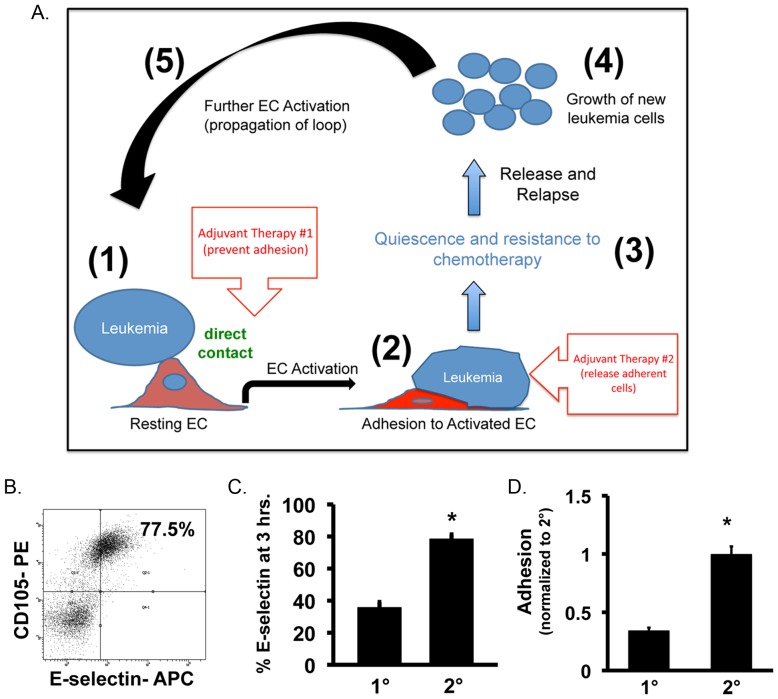
Secondary activation of ECs by leukemia cells contributes to a positive feedback loop mechanism. (**A**) Schematic of positive feedback loop demonstrating the effects of activated ECs on the survival and growth of leukemia cells. Leukemia cells induce EC activation (Step 1) leading to adhesion (Step 2). Following adhesion, leukemia cells become quiescent and are unaffected by chemotherapy (Step 3). Following removal of chemotherapy agents, leukemia cells are eventually released and begin to proliferate in a process resembling relapse (Step 4). Newly released cells can propagate the loop (Step 5). Also shown are potential adjuvant therapy sites that could be used to disrupt the loop and thus augment chemotherapy for more optimal disease treatment. (**B**) Representative flow plot showing the ability of leukemia cells to activate secondary ECs. (**C**) E-selectin levels following primary and secondary activation with KG-1 cells. * p<0.05. (**D**) Primary and secondary adhesion levels of KG-1 onto leukemia activated ECs. Values were normalized to secondary adhesion levels. * p<0.05.

Based on the finding that leukemia cells themselves act as stimuli to activate ECs, we postulated the existence of a ‘positive feedback loop’ in leukemia (Figure 6A). To show this loop exists, we investigated the ability of leukemia cells from primary co-cultures to activate secondary resting ECs. Upon re-plating released BrdU^+^ (actively proliferating) leukemia cells into secondary co-cultures, we found that ECs displayed increased E-selectin levels after 3-hours demonstrating that newly released leukemia cells preserve their ability to activate ECs (Figure 6B). Interestingly, secondary activation was significantly higher in comparison to primary activation based on E-selectin levels suggesting that leukemia cells exposed to the effects of activated ECs are somehow primed to re-initiate the process (Figure 6C). Additionally, we found that significantly more leukemia cells adhere to secondary activated ECs, presumably due to the higher levels of activation and E-selectin expression (Figure 6D). This data demonstrates that once this process is initiated, in can propagate through additional activation steps resulting in a leukemia supportive positive feedback loop.

### Leukemia cells activate primary ECs leading to leukemia cell adhesion, quiescence and resistance to chemotherapy

**Figure 7:**
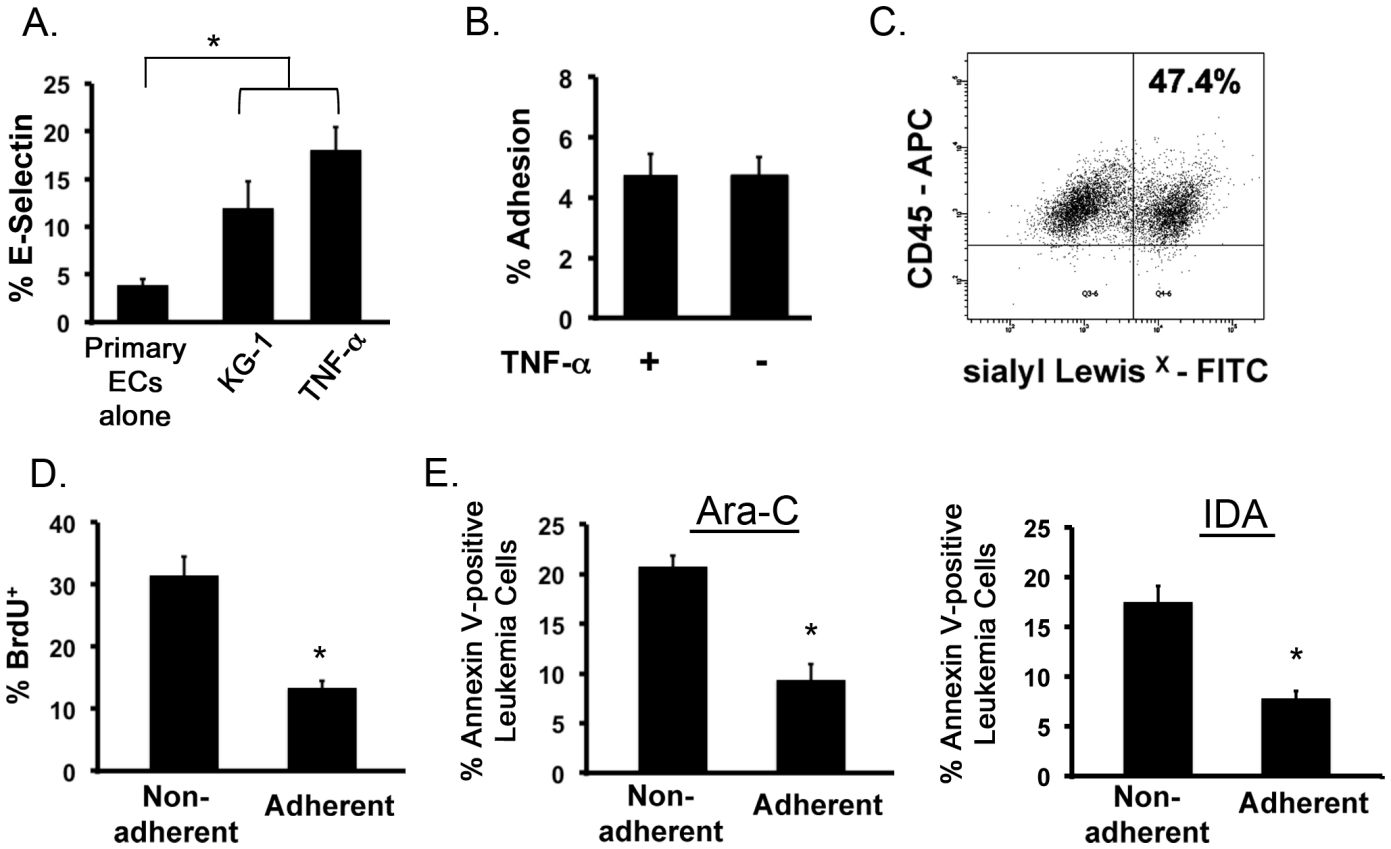
Leukemia cells activate primary ECs resulting in adhesion of leukemia cells to EC layer and protection from chemotherapy. (**A**) Percent E-selectin expression on primary ECs increases when co-cultured with KG-1 cells. E-selectin expression of resting ECs and primary ECs treated with TNF-α are negative and positive controls, respectively. * p<0.05. (**B**) The levels of KG-1 cell adhesion to activated ECs were determined in the presence and absence of TNF-α. (**C**) Representative flow cytometry plot showing expression of sialyl Lewis^X^ on the surface of KG-1 cells. (**D**) BrdU uptake by non-adherent and adherent KG-1 cells cultured with primary ECs analyzed by flow cytometry. * p<0.05. (**E**) Annexin V levels of non-adherent and adherent KG-1 cell populations co-cultured with primary ECs exposed to 200 µM Ara-C or 0.1 µM IDA for 24 hours is shown. * p<0.05.

Next, we tested whether leukemia cells were able to activate a more clinically relevant model comprising primary bone marrow-derived human ECs. To demonstrate primary EC activation, we measured the expression of E-selectin on resting ECs as well as ECs from contact co-cultures with KG-1 cells. As a positive control, E-selectin expression of TNF-α treated primary ECs was evaluated. The data showed that resting primary ECs expressed low levels of E-selectin averaging 3.9±0.5% (Figure 7A). However, addition of KG-1 cells or TNF-α to culture significantly increased E-selectin expression demonstrating that primary ECs can be activated by leukemia cells (Figure 7A). It is noteworthy that the level of activation was lower for primary ECs in comparison to HUVECs. This finding demonstrates that ECs derived from different sources have varying susceptibility to leukemia-induced activation and corroborate the known heterogeneity of EC behavior based on type [33], [34].

We then examined the ability of leukemia cells to adhere to activated primary ECs. The results were consistent with our prior findings with HUVECs and showed that leukemia cells adhere to activated primary ECs (Figure 7B). Adherence levels were similar to those observed in TNF-α treated positive controls. Similar to our activation levels, percent adhesion to primary ECs was lower in comparison to HUVECs with values of 4.7±0.6 and 16.2±1.2%, respectively, resulting in 2.4±0.3×10^5^ leukemia cells adhering to primary ECs. This finding further demonstrated that levels of adhesion directly correlate to levels of activation. To determine if adhesion also correlated to ligand expression, KG-1 cells were analyzed for sialyl Lewis^X^ levels, a major E-selectin binding ligand in settings of leukemia [35], [36], [37]. The data demonstrated that 47.9±0.6% of KG-1 cells expressed this ligand (Figure 7C). Given this high ligand expression, the data suggests that adhesion may not be tied to ligand expression and is more likely modulated by activation (i.e. E-selectin expression) levels. However, E-selectin has other known binding ligands that may participate in this process including P-selectin glycoprotein ligand-1 (PSGL-1), endoglycan, lysosome-associated membrane protein (LAMP)-1, LAMP-2 and CD44 among others [35], [36], [37], [38], [39], [40], [41]. Further analysis of these ligands may reveal their involvement in the observed adhesion. Overall, these studies demonstrate that EC activation acts as a trigger to initiate leukemia cell adhesion to primary ECs.

Based on these results, we next determined the proliferative status of adherent leukemia cells. As before, adherent and non-adherent KG-1 cells were assessed for BrdU uptake. The results showed that a significantly higher number of adherent leukemia cells were quiescent in comparison to non-adherent cells (Figure 7D). Given this quiescence, the chemotherapy protective effects of activated primary ECs was then examined. Here co-cultures of KG-1 and primary ECs were established and treated with 200μM Ara-C or 0.1μM IDA (a non-S-phasic chemotherapeutic agent). For both Ara-C and IDA treated co-cultures, the percent Annexin V positive cells was significantly higher in the non-adherent leukemia cell populations (Figure 7E). This data shows that primary ECs have the ability to protect adherent leukemia cells from chemotherapy and contribute to chemo-resistance when treated with both S-phasic and non-S-phasic agents.

## Discussion and Conclusion

In this study we demonstrate a novel mechanism wherein the inflammatory response of EC activation initiates activities that play a role in leukemia progression and relapse. We found that EC activation leads to increased leukemia cell adhesion to ECs resulting in leukemia cell quiescence and resistance to chemotherapy (Ara-C). Upon their release, leukemia cells re-enter cell cycle suggesting that this process may be involved in the high incidences of relapse. Preventing or inducing the release of adherent leukemia cells helps disrupt this protective environment, making more cells susceptible to chemotherapy, increasing overall leukemia cell killing and potentially eliminating the cellular components involved in relapse. These observations point to the importance of considering adjuvant therapies that aim to prevent this interaction and/or release already adherent leukemia cells as a way to augment the effects of current chemotherapy (see Figure 6A; adjuvant therapy insets). From our results, such adjuvant therapies may include blocking antibodies against E-selectin or other cell adhesion molecules such as P-selectin, VCAM-1, ICAM-1 and PECAM-1 based on their importance in malignant hematopoiesis [21], [26], [30], [39], [42], [43], [44]. Interestingly, VCAM-1 and ICAM-1 also demonstrate increased expression during endothelial cell activation [27]. Studies have shown that *in vivo* administration of such agents does not adversely affect animal survival leading to the possibility of using this approach as part of standard treatment however; more extensive animal studies that define antibody dosage, administration route and schedule and effect on normal hematopoiesis are essential to translate these findings to the clinic.

Our finding that leukemia cells themselves can activate ECs, and thus initiate this process, is indicative of a positive feedback loop scenario (Figure 6A). In this loop, leukemia cells produce conditions that activate ECs (Figure 6A; Step 1). In response, activated ECs increase expression of cell adhesion molecules such as E-selectin, which promote leukemia cell adhesion in a process resembling leukostasis (Figure 6A; Step 2) [26], [30]. In this attached state, leukemia cells become quiescent and are protected from standard chemotherapy (Figure 6A; Step 3). These bound cells may represent residual cellular mediators of leukemia relapse given our finding that upon release these cells re-enter cell cycle even following Ara-C treatment (Figure 6A; Step 4). Finally, we show that leukemia cells that are released from activated ECs can further activate secondary ECs to propagate the loop (Figure 6A; Step 5). The finding that secondary activation results in a more pronounced EC activation response suggests that additional cycles may contribute to heightened leukemia burden. Overall, a deleterious positive feedback mechanism is established that supports the survival of leukemia. The identification of this regulatory mechanism may help identify new therapies for leukemia that aim to disrupt this supportive process.

The observation that KG-1 and HL-60 cells promoted different kinetics of activation suggests that different AML subtypes may participate in this process to varying degrees. Our data indicates that AMLs similar to KG-1, which are phenotypically minimally differentiated early myeloblasts (M0) based on French-American-British (FAB) classification may be engaged in this process more robustly than ones similar to the promyelocytic HL-60 cells, which are more closely related to the M3 FAB classification [45]. Our findings may explain why M0 AML has been historically harder to treat. The presenting characteristics and the outcome of patients with M0 AML have been described, and a consistent feature is a low remission induction rate and short remission duration [46], [47]. Overall, M0 patients have a very poor prognosis with standard therapies and their unresponsiveness to chemotherapy often leads to treatment failure [48]. In fact, it has been postulated that nonconventional therapeutic approaches should be developed to alter the prognosis of this form of leukemia. Alternatively, the decreased ability of HL-60 cells to induce the cascade of protective events associated with EC activation may also explain the positive chemotherapy-based treatment results already observed with promyelocytic AML patients [49]. HL-60 cells do not express the t(15;17) translocation, however they are still thought to represent an M3 acute promyelocytic leukemia due to appropriate morphology and response to differentiating agents such as all trans retinoic acid (ATRA). Acute promyelocytic leukemias of this type are most often treated with ATRA, however; studies have also tested the effects of standard chemotherapy. In long-term follow-up studies of high-risk patients with promyelocytic leukemia it was found that Ara-C treatment had a profound positive effect on the prevention of relapse [49]. It should be noted that the benefits of using ATRA as a treatment for this type of leukemia suggests that EC activation does not protect cells against differentiating-inducing treatment strategies. Based on these findings, we postulate that the ability of leukemia cells to activate ECs may be a significant determinant on therapeutic outcomes following chemotherapy. In this manner, screening a patient’s cells for their activation potential (using similar *in vitro* assays described in this study) may serve as a prospective indicator of therapy outcome. Ongoing studies using primary AML samples of different sub-types and varying genetic mutations are now being performed to test this hypothesis.

This study demonstrated that leukemia cells preferentially adhere to activated ECs, conferring chemotherapy resistance, and this may be a direct result of up-regulated E-selectin levels. These results are supported by previous reports wherein rigorous *in vivo* confocal imaging was used to show that, following transplantation, leukemia cells preferentially migrated to bone marrow sites near molecularly distinct stromal cell-derived factor (SDF)-1^+^ E-selectin^+^ ECs [26]. Furthermore, others demonstrated that the perivascular endothelial region generates microenvironments that protect leukemia cells from chemotherapy [50]. Through the use of primary human AML samples, ongoing studies are now deciphering the specific cell populations that adhere to activated ECs. Our hypothesis is that repopulating CD34^+^CD38^−^ leukemia initiating stem cells (LSCs) will represent at least one sub-set of cells that preferentially adhere. The finding that the selectin ligand CD44, a glycoform of the hyaluronan receptor, plays a key role in LSC (BCR-ABL^+^) homing and engraftment to supportive microenvironmental niches *in vivo* provides support for this theory [42]. This likely scenario would identify a mechanism that would add to the growing understanding of the behavior of LSCs in relapse.

Finally, EC activation is an inflammatory response that occurs in response to factors such as infectious agents, cytokines and various pharmaceuticals [27], [51]. Inflammation is known to play a role in various solid tumor-based cancers and is now considered to be a hallmark of cancer [52]. Our findings point to a similar intimate involvement of inflammation in leukemia growth and survival, mediated through EC activation. This is an important observation as it opens new avenues for AML therapy to include those agents that prevent EC activation. Use of anti-inflammatories known to decrease EC activation such as anti-TNF-α antibodies (already used to treat rheumatoid arthritis) [53], antioxidants or glucocorticoids [54] in conjunction with chemotherapy may have significant additive effects on improving treatment outcome. Such combination treatments would prevent EC activation and diminish adhesion effects increasing leukemia cell sensitivity to chemotherapy. We are interested in developing and testing these types of combination therapies that would not only treat the existing leukemia but also prevent subsequent relapse. Ultimately, these novel strategies may be applicable to many hematological malignancies.
